# Ready, set, go! The role of organizational readiness to predict adoption of a family caregiver training program using the Rogers’ diffusion of innovation theory

**DOI:** 10.1186/s43058-023-00447-x

**Published:** 2023-06-19

**Authors:** Courtney H. Van Houtven, Connor Drake, Teri L. Malo, Kasey Decosimo, Matthew Tucker, Caitlin Sullivan, Josh D’Adolf, Jaime M. Hughes, Leah Christensen, Janet M. Grubber, Cynthia J. Coffman, Nina R. Sperber, Virginia Wang, Kelli D. Allen, S. Nicole Hastings, Christopher M. Shea, Leah L. Zullig

**Affiliations:** 1 Center of Innovation to Accelerate Discovery and Practice Transformation, Durham VA Health Care System (152), 508 Fulton Street, Durham, NC 27705 USA; 2 Department of Population Health Sciences, Duke University, Durham, NC USA; 3 Duke-Margolis Center for Health Policy, Duke University, Durham, NC USA; 4 Department of Implementation Science, Wake Forest School of Medicine, Winston-Salem, NC USA; 5 Section on Gerontology and Geriatric Medicine, Division of Internal Medicine, Wake Forest School of Medicine, Winston-Salem, NC USA; 6 Veteran’s Health Administration Central Office, Washington, DC, USA; 7 Cooperative Studies Program Coordinating Center, Veterans Affairs Boston Healthcare System, Boston, MA USA; 8 Department of Biostatistics and Bioinformatics, Duke University Medical Center, Durham, NC USA; 9 Department of Medicine & Thurston Arthritis Research Center, University of North Carolina at Chapel Hill, Chapel Hill, NC USA; 10 Center for the Study of Aging and Human Development, Duke University School of Medicine, Durham, NC USA; 11 Geriatric Research, Education, and Clinical Center, Durham VA Health Care System, Durham, NC USA; 12 Division of Geriatrics, Department of Medicine, Duke University School of Medicine, Durham, NC USA; 13 Department of Health Policy and Management, University of North Carolina at Chapel Hill, Chapel Hill, NC USA

**Keywords:** Family caregivers, Veterans, Skills training, Informal care, Implementation science

## Abstract

**Background:**

Caregivers FIRST is an evidence-based program addressing gaps in caregivers’ skills. In 2020, the Veterans Health Administration Caregiver Support Program (CSP) nationally endorsed Caregivers FIRST, offering credit in leadership performance plans to encourage all VA medical centers (VAMCs) to implement locally. This study examines the association of organizational readiness with VAMC adoption of Caregivers FIRST.

**Methods:**

In a cohort observational study, we surveyed CSP managers about their facilities’ readiness to implement using the Organizational Readiness for Implementing Change (ORIC) instrument and compared change commitment and change efficacy domains among VAMCs “adopters” defined as delivering Caregivers FIRST within 1 year of the national announcement to those that did not (“non-adopters”). Within “adopters,” we categorized time to adoption based on Rogers’ diffusion of innovation theory including “innovators,” “early adopters,” “early majority,” “late adopters,” and “laggards.” Organizational readiness and site characteristics (facility complexity, staffing levels, volume of applications for caregiver assistance services) were compared between “adopters,” “non-adopters,” and between time to adoption subcategories. Separate logistic regression models were used to assess whether ORIC and site characteristics were associated with early adoption among “adopters.”

**Results:**

Fifty-one of 63 (81%) VAMCs with CSP manager survey respondents adopted Caregivers FIRST during the first year. ORIC change commitment and efficacy were similar for “adopters” and “non-adopters.” However, sites that adopted earlier (innovators and early adopters) had higher ORIC change commitment and efficacy scores than the rest of the “adopters.” Logistic regression results indicated that higher ORIC change commitment (odds ratio [OR] = 2.57; 95% confidence interval [CI], 1.11–5.95) and ORIC change efficacy (OR = 2.60; 95% CI, 1.12–6.03) scores were associated with increased odds that a VAMC was an early adopter (categorized as an “innovator,” “early adopter”, or “early majority”). Site-level characteristics were not associated with Caregivers FIRST early adoption.

**Conclusions:**

To our knowledge, this study is the first to prospectively assess organizational readiness and the timing of subsequent program adoption. Early adoption was associated with higher ORIC change commitment and change efficacy and not site-level characteristics. These findings yield insights into the role of organizational readiness to accelerate program adoption.

**Trial registration:**

ClinicalTrials.gov, NCT03474380. Registered on March 22, 2018

Contributions to the literature

- This study is among the first to use ORIC to prospectively assess organizational readiness and subsequent adoption and timing of adoption.
- This work contributes to the understanding of determinants and downstream effects of organizational readiness.
- A robust understanding of how organizational readiness and site characteristics vary by “adopter” status based on Rogers’ diffusion of innovation theory.

## Background

With 26.4 million family and unpaid caregivers in the USA actively caring for an adult with physical limitations, more than 90% do not receive the training they needed to fulfill their caregiving role [1, 2]. In 2018, the US Centers for Disease Control and Prevention (CDC) named family caregiving as a public health priority because of the unintended negative consequences of the US health care system of long-term care that primarily relies on unpaid, untrained family members and friend caregivers [3]. Thus, identifying and implementing evidence-based models to support caregivers is a priority. The National Academies of Science, Engineering and Medicine published a landmark report in 2016 recommending the spread of evidence-based trainings for caregivers as a means of expanding supports [4]. In the USA, the September 2021 RAISE Act Initial Report to Congress also contains nine recommendations to increase access to services and supports for caregivers (among other recommendations) [5]. While caregiver education and support can improve caregiver health-related quality of life and decrease depressive symptoms and psychological burden, most caregivers do not have access to supports or training [6–12]. Expanding evidence-based caregiver supports and trainings within health systems is a promising avenue to increase supports to caregivers, because of their direct contact with caregivers. Challenges exist at a health system level to systematically identify caregivers in a patient’s electronic health record, limiting the development of standardized approaches to assess caregiver risks or their training gaps [13–15]. Nevertheless, the health system setting is where patients engage in services, so patients can help identify their caregivers. In addition, caregivers often accompany patients to visits so can navigate health system offerings and play a critical role in communicating with health care teams [16–19].

The US Department of Veterans Affairs (VA) Health Care System, the largest integrated health care system in the USA, operates the most comprehensive publicly funded caregiver support program in the country. The VA Caregiver Support Program (CSP) offers skills training, one-on-one support, respite care, peer support, and access to a caregiver support team at every VA Medical Center (VAMC) to help caregivers navigate the VA and cope with their caregiving role through counseling and referrals. The VA CSP and the VA Health Care System have sought to embrace a culture of innovation and improvement. CSP has driven the development of multiple new trainings in its 10-year existence, using input from the field staff. For example, trainings on intimate partner violence, peer support programs, and trainings that address caregiving challenges for specific diseases have been developed, disseminated, and refined [20, 21]. Yet, even in the VA Health Care System, around 50% of caregivers report they have not received the training they need [22]. Based on these persistent gaps in training, our team partnered with VA CSP in 2018 to expand evidence-based caregiver training (Caregivers FIRST, formerly iHI-FIVES [23]) to eight individual medical centers as a part of a larger implementation science research study (Function QUERI) [24]. In 2020, VA announced Caregivers FIRST as a “strong practice,” meaning that it was one of three education programs that 142 VA medical centers with CSP staffing could offer to meet annual leadership performance plans, where each medical center reports status on key performance indicators to track progress towards meeting requirements within the CSP Program of General Caregiver Supports and Services. In addition, CSP strongly encouraged each VAMC to add these “strong practice” programs to their VA regional network director and medical center director annual performance plans. Elevating such programs to leadership performance plans where there is a shared understanding across leadership and service delivery for benchmarking success could be a driver for adoption especially given that leadership is widely understood to be a key aspect of context related to implementation outcomes [25]. Additionally, VAMCs were provided implementation support based on the Replicating Effective Programs (REP) framework. This multi-pronged approach sought to accelerate adoption and compress the timeline for the diffusion of Caregivers FIRST among eligible VAMCs. REP support and inclusion in leadership performance plans were provided to all sites; thus, it is critical to understand the conditions and organizational characteristics of VAMCs wherein these investments in implementation resulted in adoption.

Inclusion of Caregivers FIRST as one of three “strong practice” programs to meet CSP performance plans may encourage adoption, but this alone may be insufficient for predicting early program implementation, whereas aspects of organizational readiness such as task knowledge, resource availability, and situational factors may also contribute to adoption [26]. Organizational readiness for change, defined as the organizational members’ psychological and behavioral preparedness to implement change, may be one important predictor of adoption decisions and subsequent implementation success [26, 27]. Implementation scientists have also conceptualized readiness for change at the organizational level reflecting collective commitment or efficacy defined as “a comprehensive attitude” associated with effective organizational change [26, 27]. A 2020 systematic review and content analysis of organizational readiness assessments found limited research evaluating the relationship between organizational readiness and implementation outcomes, highlighting the importance of ongoing research testing these relationships [28]. In a 2017 study using Organizational Readiness for Implementing Change (ORIC) among sites in a randomized controlled trial to implement evidence-based health promotion practices at low-wage worksites, change commitment declined at the intervention sites despite increases in implementation efforts. This finding may have been attributed to “change fatigue” or regression to the mean; however, adoption or timing of adoption was not evaluated as an implementation outcome [29]. There is significant interest, yet scant empirical data on how organizational readiness predicts subsequent program adoption, defined as uptake, utilization, or initial implementation [30]. For example, a 2017 systematic review of organizational characteristics that predict adoption indicates that “readiness for implementation” is a frequently reported determinant of adoption but the inconsistent use of standardized reporting criteria or validated instruments represent barriers to advancing evidence on this topic [31]. However, there is reason to believe that organizational readiness is a distinct characteristic of organizations that could be measured and predict adoption timing. Leveraging ORIC to prospectively assess its relationship with subsequent adoption could help address this persistent gap in implementation research.

Adoption of a novel program, like Caregivers FIRST, in health care settings involves a complex interplay of organizational changes that occur over time and varies by setting in “waves” wherein some organizations are apt to adopt sooner than others [32]. Moreover, time to adoption is associated with the fit of the innovation with organizational characteristics [31]. The organizational propensity of being an early adopter versus a laggard may signal different motivations, capacities, and resources. It follows that systems seeking to encourage the spread of innovations may deploy different strategies to support adoption in their organizations depending on differences in capacity, characteristics, resources, and organizational behavior [33]. Thus, understanding the prospective relationship between organizational readiness and adoption status and timing, operationalized using theoretically sound and flexible diffusion of innovation adoption categories, could fill an important evidence gap related to the translation of innovations in diverse health care contexts. Diffusion is typically graphed cumulatively and represent a retrospective view of the entirety of a process of diffusion usually applied over a longer time horizon (e.g., over years to describe adoption across a system or population). This usually begins with an initial slow rate of adoption giving way to a rapidly accelerating rate, which then slows as fewer nonadopters remain within the system in question [32]. The length of time over which this process is evaluated varies with examples in health care ranging for shorter durations (e.g., less than 3 years) [34] and others ranging for over a decade [35]. The speed at which diffusion occurs depends on innovation characteristics, contextual factors, and research question of interest. Since intensive strategies ranging from leadership credit to active implementation support were provided to VAMCs to adopt Caregivers FIRST, we sought to categorize adopting sites using Rogers’ theory based on timing of adoption across a shorter time period (12 months).

The purpose of this paper is to examine organizational readiness and subsequent program adoption status and timing of Caregivers FIRST following its announcement as a “strong practice.” To our knowledge, this study is the first to use the ORIC instrument to prospectively assess organizational readiness and subsequent adoption and timing of adoption. This work advances implementation research by examining the relationship between organizational readiness and subsequent, nation-wide program adoption in the nation’s largest integrated health system.

## Methods

### Design

Caregivers FIRST (Caregivers Finding Important Resources, Support, and Training) is an evidence-based group training for friends or family members who are caregivers of veterans. A randomized controlled trial evaluated the effectiveness of the original program, which demonstrated promise in filling caregiver training gaps and increasing an important metric of care: self-reported experience with quality of care [23]. The Veteran care recipients included in the study can be from any era of service or have any condition meriting the need for a family caregiver. The core component of the intervention is four core modules designed to promote veteran function and independence through building caregiver hands-on, coping, support-seeking, and health system navigation skills [23, 36]. The curriculum has been flexibly designed, allowing for adaptation and tailoring to increase fit to implementers and recipients while maintaining fidelity to the four core components. The modules can be delivered within the CSP or in partnership with other VAMC service lines (e.g., chaplaincy, mental health, primary care), with in-person or virtual modality options. Optional supplemental materials include six topical videos/phone scripts and post-training “booster” sessions [36].

To nationally disseminate Caregivers FIRST, our overarching implementation strategy was Replicating Effective Programs (REP) [37, 38], which is a low-cost and low-burden strategy that addresses implementation barriers and promotes intervention tailoring to fit local conditions [39, 40]. As part of REP, the study team provided training standardized implementation tools (program toolkit, curriculum, and marketing materials) [11], electronic health record documentation templates, brief technical support via email and monthly office hours, and quarterly diffusion network calls for sites implementing the program to share lessons learned. We selected REP as a bundle of implementation strategies to all VAMCs seeking to adopt Caregivers FIRST because it has been tested and empirically validated across a variety of clinical trials using robust observational and experimental designs. Secondly, REP is adaptable and can allow for standardized support that is tailored across four stages of implementation [39–43]. Our objective in this paper was not to assess the impact of REP implementation support; additional detail on the selection of strategies and operationalization of implementation support for Caregivers FIRST has been published elsewhere [11, 24].

To explore organizational readiness and adoption of Caregivers FIRST as a “strong practice” program, the study team designed a national, cross-sectional survey for a cohort observational study to be completed by CSP managers who are responsible for the administration and performance of their VAMC’s caregiver support program. Based on a 2020 systematic review, organizational readiness assessments [28, 44] have typically collected responses at the collective level of a team; however, we approached a centralized contact as a representative. Our survey was administered during a time of rapid CSP growth because of the passage of the 2018 VA Mission Act, including increased staffing to expand capacity for providing additional training and caregiver services [21]. Thus, for feasibility, we asked CSP managers, who are very familiar with staffing, workflows, resources, and capacity to report on the efficacy and commitment to change on behalf of their work unit. The purpose of the survey was to understand a participating site’s CSP program readiness to implement Caregivers FIRST (Appendix 2) as a “strong practice,” as well as assess anticipated or current barriers to implementation. The survey was approved by the Institutional Review Board of the Durham VA Health Care System and received concurrence from the VA national labor unions. We adhered to STROBE guidelines for reporting observational studies.

### Setting and participants

**Fig. 1:**
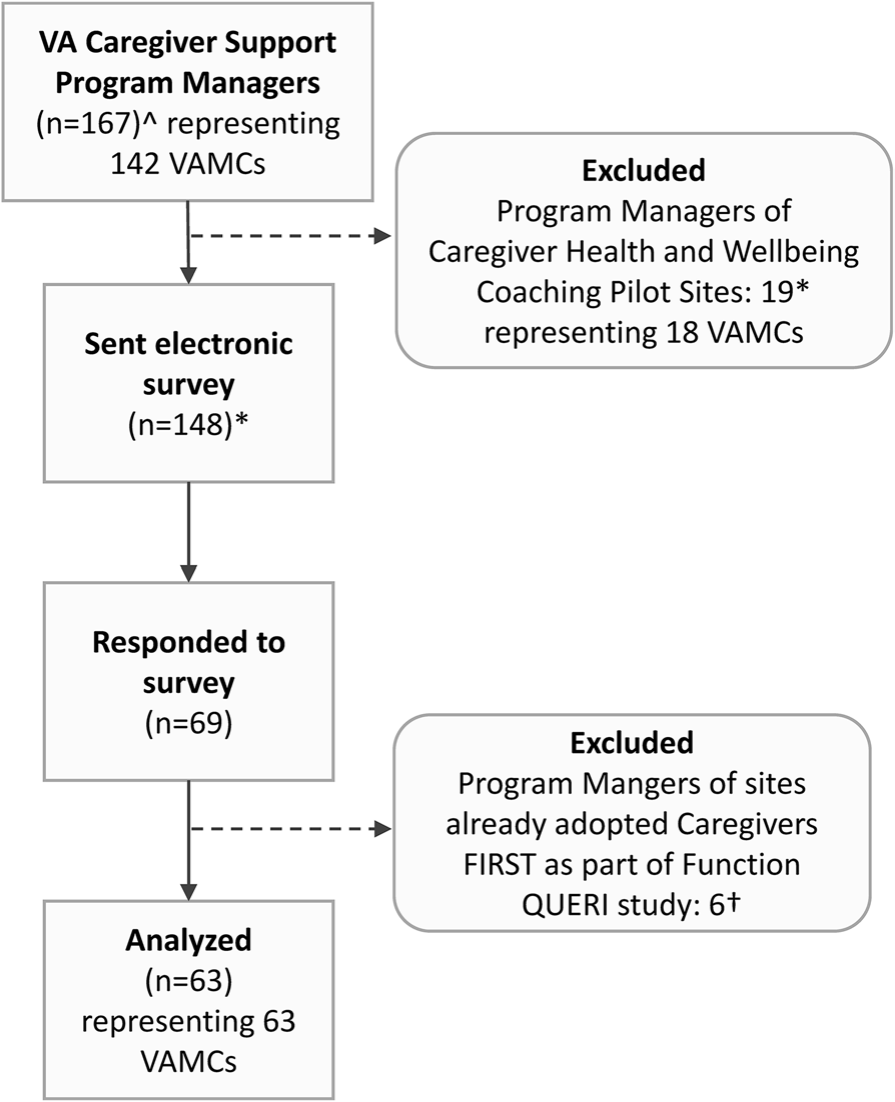
Caregivers FIRST ORIC Survey Respondents. ^^^As of January 2021, the CSP staff identified as program managers or back-ups through CSP program manager distribution list. *Excluding 18 pre-identified CSP Whole Health pilot sites, who will not be considering Caregivers FIRST as a strong practice program in FY21: VA Illiana VA HCS, St. Cloud VA HCS, VA Hudson Valley HCS, James H. Quillen VAMC, Phoenix VA HCS, Ann Arbor VAMC, VA Northern California HCS, Harry S. Truman Memorial, Salisbury - W.G. (Bill) Hefner VAMC, VA Texas Valley Coastal Bend HCS, Hershel “Woody” Williams VAMC, Southeast Louisiana VA HCS, VA Boston HCS, VA Caribbean HCS, Columbia VA HCS, Corporal Michael J. Crescenz VAMC, VA Eastern Colorado Health Care System (ECHCS), and White City or VA Southern Oregon Rehabilitation Center. ^†^Respondents who had already adopted Caregivers FIRST prior to the “strong practice” announcement were part of the Function QUERI implementation study 2018–2020

The adoption period of interest is October 2020 through September 2021, which aligned with the CSP fiscal year performance plan reporting requirements. In October 2020, Caregivers FIRST was announced nationally as a “strong practice” program. After confirming with CSP leadership that VAMCs were not intending to adopt Caregivers FIRST in the first 3 months (primarily because of staffing changes and hiring), the study team invited CSP managers nationally to participate in the survey in January 2021. The CSP program office provided a list of active CSP managers (*n* = 167) representing 142 VAMCs that receive support from CSP and undergo annual program performance assessments. Eighteen VAMCs were excluded because CSP had already designated those facilities to implement a different “strong practice” program in FY21 called Caregiver Health and Wellbeing (adapted for caregivers from veteran-focused Whole Health training) (Fig. 1).

In January 2021, the study team administered the survey electronically via VA REDCap (Research Electronic Data Capture) [45] by sending an email (with two weekly follow-up reminders) to 148 CSP managers representing the 124 eligible VAMCs. Survey respondents (*n* = 69 CSP managers, response rate of 49%) represented 69 of 124 eligible VAMCs (56%). The final analytical sample (*n* = 63 CSP manager surveys from 63 VAMCs) excluded 6 CSP manager surveys from 6 VAMCs that previously adopted Caregivers FIRST as part of the Function QUERI implementation study between April 2018 and October 2020 [24], prior to the “strong practice” announcement (Fig. 1). Additional detail comparing responders to non-responders has been provided (Appendix 1).

### Measures

#### Adoption

**Fig. 2:**
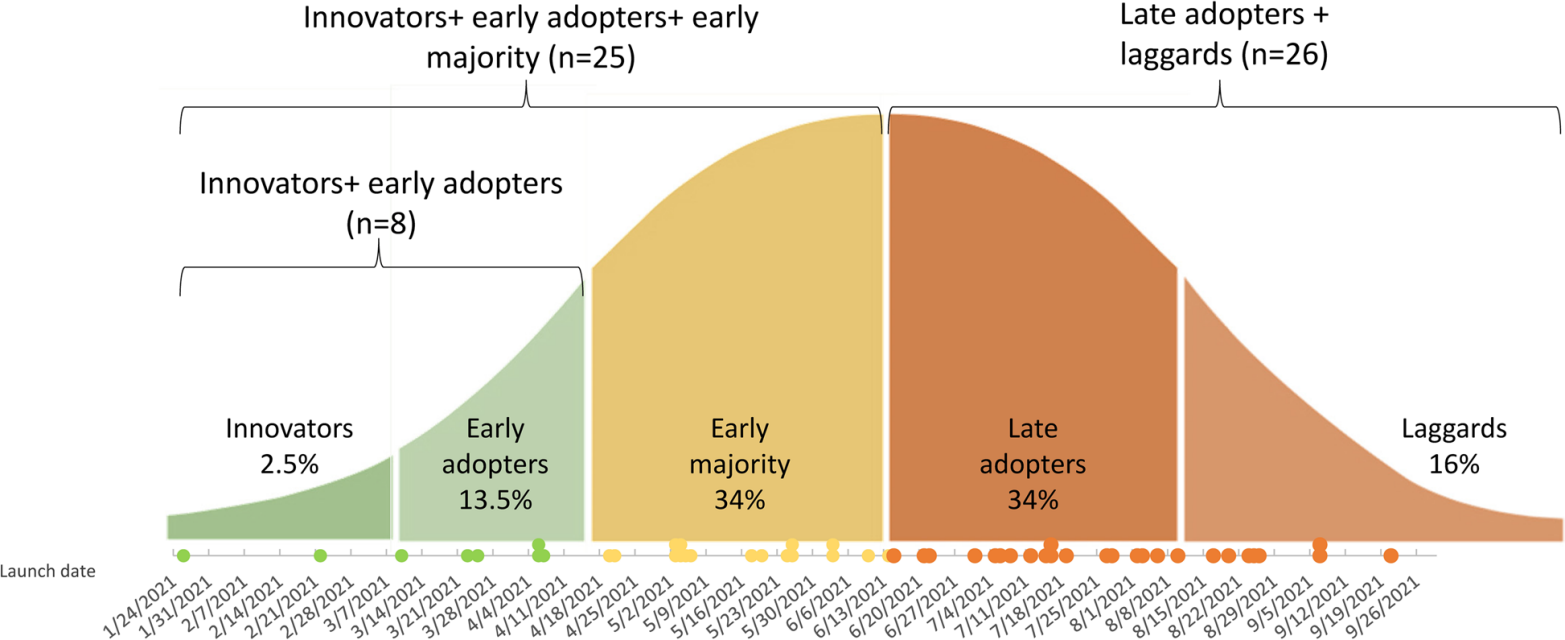
Adopter categories by program launch date for Caregivers FIRST, adopter categories are driven by Rogers’ distribution, based on launch date between 1/1/2021 and 9/30/2021. Of the sites who adopted between 1/1/2021 and 9/30/2021 (*n* = 51 sites): late adopters + laggards defined as launch between 6/15/2021 and 9/30/2021. These sites represent 51% of launchers (*n* = 26 sites). Innovators + early adopters + early majority defined as launch between 1/1/2021 and 6/14/2021. These sites represent the first 50% of launchers (*n* = 25 sites). Innovators + early adopters defined as launch between 1/1/2021 and 4/7/2021. These sites represent the first 16% of launchers (*n* = 8 sites)

Our primary outcome of interest was the Caregivers FIRST program adoption. Adoption is the intention, initial decision, or action to employ a novel intervention or practice [30]. The study team defined adoption as delivering at least one round (four classes) of Caregivers FIRST training within 12 months after the “strong practice” announcement. The study team used Rogers’ diffusion of innovation theory to further specify “adopter” categories within a 12-month period: “innovators” which is the first 2.5% of adopters, “early adopters” which is the next 13.5% of adopters, and “early majority” which is the next 34% of adopters [32, 46] (Fig. 2). The remaining 50% of adopters of comprise “late adopters” and “laggards.”

In partnership with CSP, the study team tracked VAMC adoption in a spreadsheet, including date of Caregivers FIRST program launch, as part of the CSP annual performance assessment planning. All 142 VAMCs were given access to the list of sites and the recorded launch dates and confirmed the accuracy of program launch date.

In all, 51 of 63 sites (81%) adopted within 12 months after the “strong practice” announcement (Fig. 2). The innovators + early adopters launched between 1/24/2021 and 4/7/2021. These sites combined represent the first 16% of launchers (*n* = 8 sites). An additional 17 sites adopted between 4/7/2021 and 6/14/2021 yielding the early majority group of launched sites. The innovators + early adopters + early majority sites represent the first 50% of launchers (*n* = 25 sites). Late adopters + laggards launched between 6/15/2021 and 9/30/2021, representing the remaining 51% of launchers (*n* = 26 sites).

##### Organizational readiness

We used the 12-item ORIC instrument to assess shared resolve to implement a change (change commitment domain; 5 items) and shared belief in their collective capability to make a change (change efficacy domain; 7 items) [26]. We selected this measure because previous psychometric assessment demonstrated its content adequacy and consistency for the change commitment and change efficacy scales, it is widely used, and it is associated with implementation outcomes of interest [26, 47, 48]. Each of the ORIC items is scored using a 5-point Likert scale (i.e., “disagree (1)” to “agree (5)”). The mean subscale scores ranging from 1 to 5 were calculated and presented, with higher scores indicating higher readiness.

##### Organization characteristics

Collaborating with CSP, we also developed items to assess organization characteristics that were anticipated to impact Caregivers FIRST implementation [11, 24]. Organizational, or site-level, characteristics included facility complexity, staffing, program volume/demand for caregiver assistance, and geographic region.

*Facility complexity* is derived from a VA rating categorizing every hospital based on levels of patient volume and risk, teaching and research, intensive care units, and physician specialist staffing [49]. Specifically, facility complexity ranges from 1 (the most complex with the largest levels of patient volume, patient risk, teaching and research; largest number and breadth of physician specialists; and a level 5 intensive care unit), level 2 (medium complexity), and level 3 (lowest level of complexity). High-complexity hospitals are typically urban and have the largest volume of patients and medical services [50] and therefore may also have more caregivers to serve and more staff to support caregiver programming. In addition, complexity could be related to implementation climate, capacity, and successful change. Commensurate with high volume, sites with higher complexity ratings may have additional education and training resources, including those available for continuous quality improvement relative to sites with lower complexity ratings.

*Staffing* refers to the number of full-time equivalent CSP staff hired and planned to hire based on CSP internal programmatic data from the same time period as the ORIC survey was fielded. These staff are predominantly clinical social workers and nurses. More staff hired could enhance the ability to offer new programs such as Caregivers FIRST, whereas more staff planned for hire could indicate insufficient staff currently (during the adoption year) exists to implement new programs.

*Demand for caregiver support services* is the number of unique applications submitted for the Program of Comprehensive Assistance for Family Caregivers (PCAFC). Applications are submitted by the veteran and their caregiver, and there could be multiple pathways to apply, such as from the veteran’s primary care provider, from a social worker within or outside the Caregiver Support Program or based on a recommendation of another caregiver. Number of applications could indicate demand for caregiver support services and serve as a proxy for assessing CSP volume or demand at each facility.

*Geographic region* is based on US Census Bureau geographic regions of responding VAMCs (West, Midwest, Northeast, or South) and whether a VAMC was in a rural area as classified by rural–urban commuting area codes (RUCA) from the VHA Office of Rural Health [51, 52].

#### Analyses

We used descriptive analyses for the number and proportion of facilities adopting Caregivers FIRST, types of adopters, VAMC site characteristics, and ORIC scores by domain. ORIC scores were summarized among medical centers that implemented at least one round of Caregivers FIRST training within the year after the “strong practice” announcement (“adopters”) as well as those that did not (“non-adopters”). Within “adopters,” “innovators + early adopters” and “innovators + early adopters + early majority” subgroups were compared to “late adopters” and “laggards.” As part of an exploratory analysis among the *n* = 51 sites that adopted Caregivers FIRST, we fit separate simple logistic regression models to examine the association between organizational readiness, using (1) ORIC change commitment and (2) ORIC efficacy scores; VAMC organization characteristics ((3) facility complexity, (4) staffing, and (5) demand for caregiver support services); and the binary outcome of early Caregivers FIRST program adoption defined as being categorized as an “innovator,” “early adopter,” or “early majority” or not (i.e., late adopters + laggards). Each of the five characteristics described above was examined as an independent variable of interest to determine whether it was associated with being an “innovator,” “early adopter,” or “early majority.”

## Results

Overall, among all of the 116 eligible VAMCs, 91 (78%) adopted Caregivers FIRST in the 12 months following the announcement of Caregivers FIRST as a “strong practice.” Of the subset of VAMCs who responded to the ORIC survey (*n* = 63), 51 (81%) adopted Caregivers FIRST as a “strong practice” and 12 (19%) were non-adopters during the same 12-month period (see Table 1).

**Table 1:** VAMC characteristics overall and by adoption status

	Total (*n* = 63)	Non-adopters (*n* = 12)	Adopters (*n* = 51)	Adopter sub-categories		
				Late adopters + laggards^a^ (*n* = 26)	Innovators + early adopters + early majority^b^ (*n* = 25)	Innovators + early adopters^c^ (*n* = 8)
*Organizational readiness, median (IQR)*						
ORIC change commitment	4.40 (1.60)	4.50 (1.2)	4.40 (1.60)	3.90 (1.60)	4.60 (1.00)	4.90 (1.40)
ORIC change efficacy	3.71 (0.86)	3.79 (0.79)	3.71 (1.00)	3.71 (1.00)	3.85 (0.71)	4.43 (1.07)
*Facility complexity level, n (%)*						
1—high complexity	29 (46)	5 (42)	24 (47)	12 (46)	12 (48)	3 (38)
2—medium complexity	16 (25)	2 (17)	14 (27)	*6* (23)	8 (32)	3 (38)
3–low complexity^d^	18 (28)	4 (33)	13 (25)	*8* (31)	5 (20)	2 (25)
*CSP staff, median (IQR)*						
Total staffing	9 (4)	10 (8)	9 (4)	9 (4)	9 (2)	8 (5)
*CSP unique applications, median (IQR)*	339 (388)	292 (510)	341 (381)	372 (393)	315 (245)	320 (195)
*Geographic region, n (%)*						
West	8 (13)	1 (8)	7 (14)	5 (19)	2 (8)	1 (13)
Midwest	17 (27)	2 (17)	15 (29)	8 (31)	7 (28)	2 (25)
Northeast	15 (24)	5 (42)	10 (20)	2 (8)	8 (32)	4 (50)
South	23 (37)	4 (33)	19 (37)	11 (42)	8 (32)	1 (13)
*Rural facility, n (%)*	7 (11)	2 (17)	5 (10)	2 (8)	3 (12)	1 (13)

^a^Defined as launch after 6/14/2021. These sites represent the second 50% of launchers (*n* = 26 sites)

^b^Defined as launch between 1/1/2021 and 6/14/2021, inclusive. These sites represent the first 50% of launchers (*n* = 25 sites)

^c^Defined as launch between 1/1/2021 and 4/7/2021, inclusive. These sites represent the first 16% of launchers (*n* = 8 sites)

^d^Includes one site in the “non-adopter” category that was excluded from a facility rating because it did not classify as having a rating

### VAMC characteristics

The percentage of high complexity level sites was larger among “adopter” (47%) than non-adopter sites (42%), and “adopter” sites had a median of 341 PCAFC applications compared to 292 applications among “non-adopter” sites. The largest proportion of “adopter” sites was in the south (37%), whereas the largest proportion of “non-adopter” sites was in the northeast (42%). About 10% of “adopter” sites were rural facilities, compared to 17% of “non-adopter” sites. Specifically, “innovators + early adopter” and “innovators + early adopters + early majority” VAMCs were more prevalent in the northeast and less prevalent in the south.

### Individual ORIC items

**Fig. 3:**
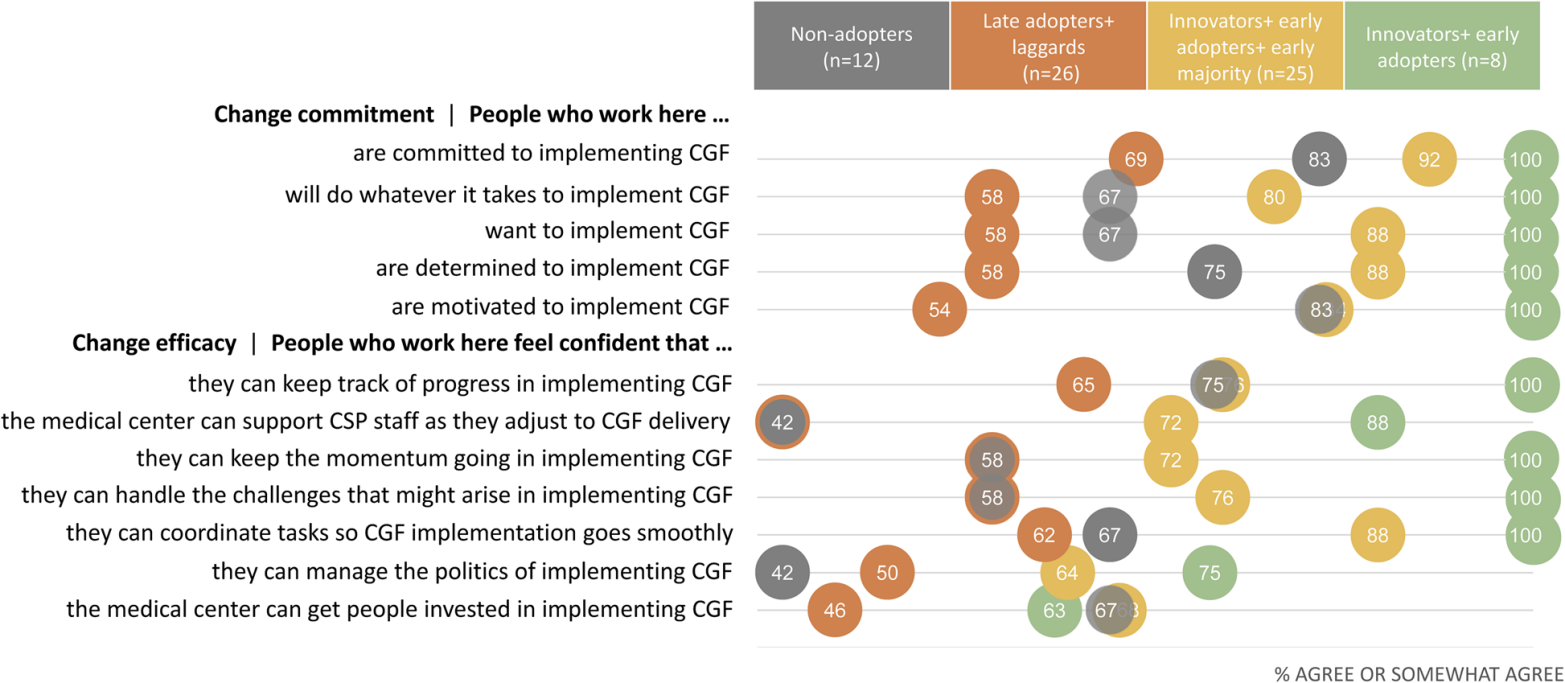
Proportion of VAMCs across “non-adopters” and “adopter” categories who agree/somewhat agree with ORIC items. *Abbreviations*: CGF, Caregivers FIRST; CSP, Caregiver Support Program. Adopter categories are driven by Rogers’ distribution, based on a launch date between 1/1/2021 and 9/30/2021. Non-adopters defined as sites that did not launch between 1/1/2021 and 9/30/2021, *n* = 12 sites. Of the sites who adopted between 1/1/2021 and 9/30/2021 (*n* = 51 sites): late adopters + laggards defined as launch between 6/15/2021 and 9/30/2021. These sites represent 51% of launchers (*n* = 26 sites). Innovators + early adopters + early majority defined as launch between 1/1/2021 and 6/14/2021. These sites represent the first 50% of launchers (*n* = 25 sites). Innovators + early adopters defined as launch between 1/1/2021 and 4/7/2021. These sites represent the first 16% of launchers (*n* = 8 sites)

For 4 of 5 individual *change commitment* items, the percentage of CSP managers who agreed or somewhat agreed with each item was relatively similar (within 4 percentage points) for managers from “adopter” and “non-adopter” sites (Fig. 3). A notably higher proportion of CSP managers from “non-adopter” sites agreed that people who work at their VAMC are motivated to implement Caregivers FIRST (83% of “non-adopter” sites vs. 69% of “adopter” sites). In contrast, a higher proportion of CSP managers from sites that adopted Caregivers FIRST agreed or somewhat agreed with most (5 of 7) *change efficacy* items. The difference between “adopter” and “non-adopter” responses was noticeable for two items: (1) “people who work here feel confident that the medical center can support CSP staff” and (2) “people who work here feel confident that they can manage the politics of implementing Caregivers FIRST.” For both of these items, 58% of CSP managers from “adopter” sites agreed or somewhat agreed, compared to 42% of managers from “non-adopter” sites.

“Innovators + early adopters” consistently agreed or somewhat agreed with items across both *change efficacy* and *change commitment* items with the exception being the “the medical center can get people invested in implementing” item. This was true to a lesser extent among the “innovators + early adopters + early majority” sites (the remaining first half of “adopters”). Notably, the “innovator + early adopters + early majority” sites (*n* = 25) had a higher percentage of managers who gave agree or somewhat agree responses across all ORIC items when compared to “non-adopter” and “late adopters + laggards” sites.

### Association between organizational readiness and organization characteristics with early Caregivers FIRST program adoption among “adopter” sites

In exploratory simple logistic regression analyses among the *n* = 51 adopter sites, there were no statistically significant associations between VAMC characteristics related to size (i.e., complexity level, staffing, or volume of PCAFC applications) and timing of Caregivers FIRST program adoption (first 50% to adopt vs. last 50% to adopt) as defined by Rogers’ diffusion of innovation theory (all *p* > 0.05; Table 2). Higher ORIC change commitment (odds ratio [OR] = 2.57; 95% confidence interval [CI], 1.11–5.95)] and ORIC change efficacy (OR = 2.60; 95% CI, 1.12–6.03) scores were associated with increased odds that a VAMC was categorized as an “innovator + early adopter + early majority.”

**Table 2:** Simple logistic regression model results of ORIC and site characteristic associations with early Caregivers FIRST program adoption among “adopter” sites

	Number	Innovators + early adopters + early majority^a^		*P*-value
		OR	(95% CI)	
ORIC change commitment	51	2.57	(1.11–5.95)	0.03
ORIC change efficacy	51	2.60	(1.12–6.03)	0.03
Facility complexity level				
1a, 1b, 1c	24	1.07	(0.35–3.27)	0.90
2, 3, Excl	27	1.00	Ref	
Unique applications	51	1.00	(0.99–1.00)	0.58
Total CSP staff	50	1.06	(0.91–1.23)	0.45

*CI* Confidence interval, *OR* Odds ratio

^a^Defined as launch between 1/1/2021 and 6/14/2021, inclusive. These sites represent the first 50% of launchers (*n* = 25 sites)

## Discussion

This study advances our understanding of the role of organizational readiness to support successful program adoption. Adoption, the decision of an organization or a community to commit to and initiate an evidence-based intervention, is a critical implementation outcome. Moreover, successful dissemination of innovative programs requires an understanding of how and why organizations adopt. This includes understanding how latent and theoretically sound determinants (e.g., organizational characteristics) influence adoption; however, this area of research is underdeveloped [31]. Adoption, by definition, is a range of activities, decisions, and intentions related to committing to a novel program. We operationalized adoption as the initiation of Caregivers FIRST via delivery of the program as part of routine care; however, future research is needed on how predictors of adoption may vary depending on how adoption is operationalized (i.e., the initial decision to implement vs. actual initiation of a novel program). By prospectively assessing organizational readiness as a theoretically sound, latent construct (i.e., a characteristic that cannot be directly observed) using the ORIC measure and observing subsequent adoption status and timing of Caregivers FIRST, we obtained insights into the characteristics of VAMCs that are conducive to the spread of an innovation. In addition to informing our understanding about readiness for implementation in the context of the Caregivers FIRST, this work also contributes to the understanding of determinants and downstream effects of organizational readiness. Specifically, this research responds to calls within the field to better bridge the gap between organizational readiness as a theoretical construct and measurable factors that are of importance to a particular set of implementation processes [28]. In this case, adoption and timing of adoption were operationalized as implementation outcomes of interest for leadership interested in the dissemination of Caregivers FIRST in the largest integrated health system in the USA.

Given similarities in “adopters” and “non-adopters” ORIC scores, our findings suggest organizational readiness may be an important measurement but is likely to be insufficient to predict adoption on its own. It is also possible, and consistent with theory [48], that ORIC scores are dynamic. In this case, the readiness scores of later adopters may have increased, if measured again, closer to the time that they adopted Caregivers FIRST and began implementation. In either case, the specifics of readiness as a necessary condition for program implementation warrants further investigation. For example, it could be that there is a threshold on the ORIC or a minimum score on a sub-scale of the ORIC that is important, such as change efficacy. Focusing on this subscale in other applications could reveal readiness as a precondition to adoption.

Assessing ORIC scores clarifies readiness to implement our specific innovation, Caregivers FIRST. The facility factors (complexity and staffing), however, did not illuminate reasons for readiness. It is possible that additional facility factors, including an organization’s overall implementation climate and context also contribute to a site’s decision and ability to adopt a new program (e.g., organization’s culture reward and support for innovation, capacity to support innovation, leadership support, sufficient resources). Also, how might past experiences adopting clinical programs affect readiness for a new program? For example, it is possible that lessons from prior experiences shape perceptions about change efficacy for a given implementation effort. When examining the subscales descriptively, it is noteworthy that non-adopter sites reported high levels of motivation and commitment but the lowest, on average, when asked about institutional support and politics. These findings suggest that certain aspects of organizational readiness represent conditions that must be met in order for adoption to occur. Additional research is needed on how these different dimensions of organizational readiness interact with each other and other external factors or characteristics of the organization.

Organizational readiness was associated with timing of adoption based on categories defined using Rogers’ diffusion of innovation theory in unadjusted models. Both ORIC domains were associated with being categorized as an “innovators + early adopters + early majority” VAMC sites in the unadjusted, simple logistic regressions models. There were no other VAMC characteristics associated with early adoption of Caregivers FIRST. We observed the differences between the ORIC scores of “innovators + early adopters” and “innovators + early adopters + early majority” sites when compared to the latter half “adopter” sites (i.e., late adopters + laggards). These findings suggest that organizational readiness may be a driver for timing of adoption, or spread, of an innovation that is distinct from other observable characteristics including complexity, program volume like PCAFC applications, or even staffing. In other words, organizational readiness is conceptually distinct. We did not find evidence that observable organizational characteristics were associated with adoption timing. This finding has implications for leadership and policymakers interested in identifying organizational-level factors that are modifiable and associated with the rapid uptake of an innovation or novel training program like Caregivers FIRST.

Our finding that higher ORIC scores were associated with being a VAMC site that more rapidly adopted Caregivers FIRST could be attributed to multiple factors. Success in adoption may be related to a culture of innovation, availability of appropriate resources, and/or acquisition of new skills needed to deliver the program. While ORIC appears to have been successful in capturing these dimensions among early adopters defined by the diffusion of innovation thresholds, it was not adequate for differentiating between “adopters” and “non-adopters.” Given that over 80% of the VAMCs in the study sample adopted Caregivers FIRST, the designation of Caregivers FIRST as a “strong practice” with corresponding performance credit may have been sufficient to ensure eventual adoption regardless of readiness. However, this policy context may have highlighted the unique value of ORIC to measure the dynamic aspects of readiness related to adoption, thereby differentiating between VAMCs that adopted Caregiver FIRST to “check the box” for performance credit and those that were prepared and motivated to rapidly deploy. At the same time, we know that dynamic staff changes occurred over the past 5 years at CSP sites nationally. Sites have also been tasked to launch and integrate a number of different clinical initiatives as the national CSP program expanded and increased field staffing during this time period. The increase in initiatives may have contributed to staff feeling overwhelmed in addressing competing priorities. Together, these examples demonstrate that past experience with innovation may have both positive and negative impacts on adopting a new clinical innovation. To this end, our findings suggest that organizational readiness, as captured by ORIC, may be an important but incomplete proxy for organizational readiness to adopt Caregivers FIRST.

These findings should be interpreted through the lens of several study design limitations. First, response rate was 49%, and adoption rates were slightly higher among responders to the survey than non-responders. Most (81%) of the responding sites adopted Caregivers FIRST and similarly, 75% of eligible non-responding sites adopted. Responding sites were also less likely to be high-complexity facilities when compared to eligible non-responders among other differences in geographic location and staffing. In particular, the observed differences between non-responding sites and responding sites’ adoption rates, though modest, may bias our results. However, by comparing the differences between responders and non-responders, findings can be better contextualized and support interpretation. Second, we have a fairly low sample size, 63 responding sites, so our ability to do statistical modeling was limited and due to high rates of adoption were unable to fit adjusted models. Still, our data are unique and provide important information through descriptive analyses. Third, the sample size and relatively short duration of the period during which adoption was measured is atypical of studies that categorize adoption using Rogers’ diffusion of innovation classifications. Fourth, previous psychometric assessments of the ORIC measure involved multiple respondents per organization, whereas our study included one respondent per organization. Although our study is not the first to do so, we are not aware of psychometric assessments of the ORIC involving one respondent per organization [29]. Finally, we chose to apply Rogers’ adopter categories across sites that adopted. This decision was informed by scrutiny of the term “laggard” and its conceptualization as being 16% of a system’s population being misleading because for many interventions [53], including mental health provider practice patterns, “non-adopters” are the majority [54]. For this reason and because most sites were adopters, we did not categorize non-adopters which should be kept in mind when interpreting our results.

There are also unique study features to highlight. Our team chose to have ORIC be reported from a single perspective per VAMC, CSP managers, instead of a whole team and other diverse perspectives (e.g., patients and caregivers). We made this decision for the feasibility of assessment in consultation with ORIC developers (CS). There are advantages and disadvantages to this approach. Disadvantages include that diversity of opinions may not be well represented and having one respondent does not allow us to triangulate differences in perspectives within an organizational unit. In other words, results may have differed if we had the perspective of multiple roles within the organization (e.g., a single respondent is limited in their ability to report shared resolve). Advantages include the feasibility of data collection and speed of response, which is crucial for informing implementation efforts in near real time. Measurement of organizational readiness may be influenced based on the unit of analysis, and little is known about the extent to which reported readiness differs based on the number, role, and nature of the respondents (e.g., there may be implications for how a CSP manager perceives organizational readiness depending on their experience in the role). We cannot determine whether this a limitation that may have systematically biased results or enabled greater measurement precision. Middle managers are understood to be key actors in facilitating the implementation of evidence-based programs [55] and since CSP managers have unique insights on the organization’s readiness for implementation as they are likely closest to internal dynamics that may impact Caregivers FIRST adoption. Future research should focus on the trade-offs between these data collection strategies to inform best practices on the assessment of organizational readiness in diverse contexts to balance considerations related to feasibility and rigor. Obtaining additional, outside perspectives could reflect unknown facility-wide barriers or facilitators that may have affected the ability to adopt. Specifically, future research should investigate organizational readiness from additional perspectives using a larger sample from contexts that are both internal and external to the organization or facility of interest. Doing so would be consistent with best practices of multi-level intervention research and has implications for intervention design [56] and selection of implementation strategies across multiple ecologic levels to increase adoption, implementation, and sustainability [57]. In addition, obtaining multiple perspectives would allow us to measure whether misaligned perspectives regarding their organizations’ readiness within an organization are associated with implementation outcomes of interest such as adoption.

We sought to address a persistent challenge in the field, namely, that the development of implementation science frameworks has outpaced the development of validated measures [58]. We selected ORIC because it has undergone prior psychometric assessment and s widely used. While measuring readiness to change is critically important, there are other factors worth considering. Our findings suggest that among candidate organizational characteristic predictor variables including staffing, CSP demand, and facility complexity, organizational readiness was the only predictor of adoption timing, or the diffusion of Caregivers FIRST in a nation-wide health care system, which is an important consideration for anticipating the rate of adoption for other evidence-based interventions. Given the lack of validated contextual measures or site characteristics that predict adoption, we may be able to use ORIC as a proxy of other unobserved contextual factors, including unobserved organizational characteristics to anticipate adoption timeliness. However, these findings are limited by the fact that only a few contextual factors were examined. Future research should explore additional, different potential determinants of adoption that can further enrich our understanding of the organizational conditions that facilitate timely adoption.

## Conclusions

This study bridges the gap between organizational readiness as a theoretical construct and measurable factors that are of importance to the adoption of Caregivers FIRST, an evidence-based family caregiver skills training program. We found that most VAMCs (81%) adopted Caregivers FIRST within 1 year of announcing it as a “strong practice” and placing leadership performance credit for its adoption in the Veterans Health Administration, the largest integrated health care system in the USA. When examining organizational readiness, facility complexity, staffing, and demand for caregiver services, we found that in simple logistic regression analyses, change commitment and efficacy ORIC domains were the only predictors of adoption timing using thresholds defined by Rogers’ diffusion of innovation theory. In doing so, this work provides empirical support to extend implementation science theory related to the conditions and organizational characteristics that support timely translation of evidence-based programs. Future research should recognize implementation as a dynamic process by assessing organizational readiness immediately preceding the “go-live” of implementation or assessing changes in readiness between early phases of the implementation process, collecting readiness data from diverse internal and external perspectives, and exploring the organizational-level determinants that may interact with readiness.

## Data Availability

Not applicable.

## Appendix 1

**Table 3:** Organizational characteristics and adoption status by responding eligible sites and non-responding eligible sites

	Total eligible^a^ (*n* = 116)	Survey sample (*n* = 63)	Not included in survey sample (*n* = 53)
*Adoption status FY21, n (%)*			
Adopted	91 (78)	51 (81)	40 (75)
Did not adopt or missing^b^	25 (22)	12 (19)	13 (25)
*Facility complexity level, n (%)*			
1—high complexity	63 (54)	29 (46)	34 (64)
2—medium complexity	22 (19)	16 (25)	6 (11)
3—low complexity^d^	31 (27)	18 (28)	13 (25)
*CSP staff, median*			
Total staffing	10	9	11
*CSP unique applications, median*	357	339	390
*Geographic region, n (%)*			
West	23 (20)	8 (13)	15 (28)
Midwest	28 (24)	17 (27)	11 (21)
Northeast	24 (21)	15 (24)	9 (17)
South	41 (35)	23 (37)	18 (34)
*Rural facility, n (%)*	12 (10)	7 (11)	5 (9)

^a^Sites not eligible included *n* = 8 Function QUERI 1.0 sites and *n* = 18 Whole Health sites. Sites not included in the survey sample are eligible sites that did not respond to the survey

^b^In fiscal year (FY) 21, sites self-reported their Caregivers FIRST launch date and left missing if they either did not adopt or did not report their launch date

## Appendix 2

**Table 4:** ORIC survey items

1	2	3	4	5				
Disagree	Somewhat disagree	Neither agree nor disagree	Somewhat agree	Agree				
A. People who work here feel confident that the medical center can get people invested in implementing Caregivers FIRST				1	2	3	4	5
B. People who work here are committed to implementing Caregivers FIRST				1	2	3	4	5
C. People who work here feel confident that they can keep track of progress in implementing Caregivers FIRST				1	2	3	4	5
D. People who work here will do whatever it takes to implement Caregivers FIRST				1	2	3	4	5
E. People who work here feel confident that the medical center can support CSP staff as they adjust to Caregivers FIRST delivery				1	2	3	4	5
F. People who work here want to implement Caregivers FIRST				1	2	3	4	5
G. People who work here feel confident that they can keep the momentum going in implementing Caregivers FIRST				1	2	3	4	5
H. People who work here feel confident that they can handle the challenges that might arise in implementing Caregivers FIRST				1	2	3	4	5
I. People who work here are determined to implement Caregivers FIRST				1	2	3	4	5
J. People who work here feel confident that they can coordinate tasks so Caregivers FIRST implementation goes smoothly				1	2	3	4	5
K. People who work here are motivated to implement Caregivers FIRST				1	2	3	4	5
L. People who work here feel confident that they can manage the politics of implementing Caregivers FIRST				1	2	3	4	5

## Abbreviations

Caregivers FIRST: Caregivers Finding Important Resources, Support, and Training
CDC: Centers for Disease Control and Prevention
CGF: Caregivers FIRST
CI: Confidence interval
CSP: Caregiver Support Program
FTE: Full-time equivalent
Function QUERI: Function and Independence Quality Enhancement Research Initiative
FY: Fiscal year
OR: Odds ratio
ORIC: Organizational Readiness for Implementing Change
REP: Replicating Effective Programs
PCAFC: Program of Comprehensive Assistance for Family Caregivers
RAISE: Recognize, Assist, Include, Support, and Engage Family Caregivers Act of 2017
REDCap: Research Electronic Data Capture
VA: Veterans Administration
VAMC: VA Medical Center
